# Hydrogen sulfide treatment alleviated ventilator-induced lung injury through regulation of autophagy and endoplasmic reticulum stress

**DOI:** 10.7150/ijbs.38315

**Published:** 2019-11-08

**Authors:** Xiaoli Ge, Jian Sun, Aihua Fei, Chengjin Gao, Shuming Pan, Zengbin Wu

**Affiliations:** 1 Emergency Department, Xinhua Hospital, Shanghai Jiao Tong University School of Medicine, Shanghai, China; 2 Cardiology Department, Xinhua Hospital, Shanghai Jiao Tong University School of Medicine, Shanghai, China

**Keywords:** ventilator-induced lung injury, hydrogen sulfide, inflammation, oxidative stress, autophagy

## Abstract

Mechanical ventilation has significant therapeutic benefits, but it may cause or aggravate lung injury, which is called ventilator-induced lung injury (VILI). Endogenous hydrogen sulfide (H2S) has roles including regulating inflammation, and promoting vasodilatation; it also exhibits anti-oxidative stress and anti-fibrosis effects. H2S has been reported to alleviate lung injury, but the effects and mechanism of H2S on VILI remain unclear. The present study established a rat model of VILI and treated them with H2S, then measured the changes in respiratory function indicators, lung tissue histopathology, and oxidative, inflammatory, and apoptotic indicators. The effect of H2S on autophagy in the VILI model and the involvement of endoplasmic reticulum (ER) stress were also investigated. To further explore the mechanism, L2 alveolar epithelial cells were treated with cyclic strain to mimic mechanical strain along with the H2S donor NaHS, and the involvement of the NF-κB/MAPK signaling pathway was examined. The results showed that H2S significantly alleviated VILI and inhibited the inflammation and oxidative stress induced by VILI. H2S also significantly reduced autophagy and ER stress in rats. The phosphorylation of IRE1α, PERK and eIF2α and the expression of nuclear ATF4, and GADD34 in L2 cells were all significantly reduced with NaHS. Nuclear NF-κB p65, MAPK p38, JNK, and ERK were all activated by cyclic strain, but inhibited by the ER stress inhibitor 4-PBA or NaHS. Our findings revealed that H2S treatment alleviated VILI by regulating autophagy and ER stress, and the PERK/eIF2α/ATF4/GADD34 and NF-κB/MAPK pathways were involved in the underlying mechanism.

## 1. Introduction

The greatest progress in the treatment of acute respiratory diseases has been mechanical ventilation. While use of mechanical ventilation has significant therapeutic benefits, improper use of this treatment (such as excessive usage time or incorrect parameter settings), may lead to or aggravate damage to the patient's lung tissue through various mechanisms, which is called ventilator-induced lung injury (VILI)^1^. To improve the therapeutic outcomes of patients who require mechanical ventilation, clarification of the mechanism of VILI and the development of new approaches for its prevention warrant further study.

In recent years, endogenous hydrogen sulfide (H_2_S) has been known as an important gas signaling molecule^2^,^3^. High concentration of H_2_S can bind to cytochrome oxidase C in mitochondria, inhibiting oxidative phosphorylation of the respiratory chain, damaging energy metabolism, and eventually leading to cell death. The physiological concentration of H_2_S, on the other hand, is essential to our bodies. Endogenous H_2_S has been confirmed to regulate inflammation^4^ and promote vasodilatation^5^, as well as exhibit anti-oxidative stress^6^ and anti-fibrosis effects. In rat acute lung injury (ALI) models induced with various factors (such as lipopolysaccharide, lung ischemia-reperfusion), exogenous administration of an H_2_S donor (sodium hydrosulfide) significantly alleviate the lung injury. Inhalation of H_2_S gas inhibits the systemic inflammatory response and improves survival in ALI or VILI^7^,^8^. H_2_S also inhibits the pulmonary inflammatory response and alveolar epithelial cell apoptosis in a hyperventilated lung injury mouse model^8^. Nevertheless, the exact mechanism of the protection exerted by H_2_S on ALI is still unknown.

Studies have found that both inflammation and reactive oxygen species (ROS) may contribute to the pathology of VILI. Under mechanical ventilation, inflammatory cytokines may be released into lung tissue, which activate multiple inflammatory signal transduction pathways^9^. Inflammatory cytokines released from different sources, such as inflammatory infiltrate, then enter the local blood circulation and cause direct damage, eventually leading to organ failure. Numerous studies have confirmed the involvement of a variety of inflammatory factors in VILI, including TNF-α and soluble mediators of pulmonary lipids^10^,^11^. Mechanical stretching could directly produce ROS^11^, which causes injury to DNA and protein, exacerbating ALI^12^.Anti-oxidant treatment has been shown to effectively reduce the degree of VILI^13^. These findings have raised questions about whether H_2_S treatment can regulate inflammation and oxidative stress, thus alleviating VILI.

Autophagy, a conserved biological process, mainly includes lysosomal degradation of intracellular substances. Autophagy plays a dual role in cell survival. For instance, autophagy protein can protect bronchial epithelial cells from hyperoxic exposure^14^; Carbon monoxide can also protect lung epithelial cells from hyperoxia by induction of autophagy^15^. However, excessive activation of autophagy may promote cell death, which is called “autophagic cell death”^16^. Therefore, the role of autophagy in cell survival depends on the stimulus and cell types 14,17. However, the involvement of autophagy in VILI and its exact mechanism remain unclear. The endoplasmic reticulum (ER) is the main assembly site for nearly all membrane proteins, where new proteins are folded and assembled into complex structures through covalent modification. Glucose deprivation, abnormal calcium regulation, viral infection, environmental toxins, hypoxia, oxidative damage, and other stress factors can all trigger physiological disorders of the ER, producing unfolded or misfolded proteins. This pathological process is called ER stress^18^. Many studies showed that ER stress is involved in the induction of autophagy^19^. ER stress inducers or hypoxic conditions can activate autophagy by inhibition of the AKT/TSC/mTOR pathway in mouse embryonic fibroblasts^20^. ER stress causes the degradation of unfolded proteins by molecular chaperones, which is called the unfolded protein response (UPR)^21^. GRP78 and GRP94 are the main markers of ER stress, and the PERK/eIF2α/ATF4 pathway is essential in the UPR^22^. The possible involvement of ER stress and autophagy in VILI piqued our curiosity.

Therefore, the present study was designed to explore the effects of H_2_S treatment on VILI, including respiratory function indicators, lung tissue histopathology, oxidative, inflammatory, and apoptotic indicators. We used an animal model of VILI to study the effect of H_2_S, and then explored its underlying mechanism using *in vitro* experiments. The effect of H_2_S on autophagy in the VILI model and the involvement of the PERK/eIF2α/ATF4/GADD34 and NF-κB/MAPK pathways were investigated.

## 2. Materials and methods

### Animals and study design

Sprague-Dawley rats (10 weeks, 250-300 g, male) were obtained from the Xinhua Hospital. The animals were housed in Shanghai Jiaotong University School of Medicine at a constant temperature of 25±2°C, relative humidity of 41%, and 12 h:12 h light/dark cycle. All the animals had free access to water and food. The experiment followed the principles of the Bio-ethic Committee of Xinhua Hospital for the care and use of laboratory animals, as well as the “Guide for the Care and Use of Laboratory Animals” (NIH Publication No. 85-23, revised 1996).

To measure the effects of H_2_S on VILI, animals were randomly assigned into five groups (n = 30): Control, Sham, VILI, H_2_S, and VILI + H_2_S. There were no differences in age or weight among groups. Ten rats were used for BALF measurement; ten rats were used for histology studies; ten rats were used for ELISA, western blot and oxidative products and enzymes studies. After rats were anesthetized by pentobarbital (50 mg/kg, i.p.) and fentanyl (0.05 mg/kg, i.p.), rats were fixed on a pad, which can maintain the body temperature of rats at 37°C. Supplementary anesthetic treatments at one third of the initial dose were given every 45 min. Rats of Control received no treatment. Rats in the Sham group were intubated and ventilated with normal tidal volume (6 ml/kg) using a ventilator (RWD407, RWD Life Science Co., Ltd., San Diego, CA, USA). Rats in the VILI and VILI + H_2_S groups were ventilated with high tidal volume (20 mk/kg) using the ventilator, as previously described 23,24. Rats in the VILI group were ventilated with air; rats in the VILI + H_2_S groups were ventilated with air supplemented with 80 ppm H_2_S for 4 h. Rats in the H_2_S group were ventilated with air supplemented with 80 ppm H_2_S at a normal tidal volume using a ventilator (RWD407, RWD Life Science Co., Ltd.) for 4 h. The concentration of H_2_S was monitored with a Hydrogen Sulfide Analyzer (Model Jerome 631-X, Arizona Instruments, Chandler, AZ, USA). When these treatments were complete, rats were sacrificed through cervical dislocation.

To measure the involvement of autophagy in the pathology of VILI, rats were randomly assigned to four groups: Control, VILI, VILI + 3-methyladenine (3-MA), and VILI + CLQ groups. 3-MA, a class III PI3K inhibitor, is usually used to selectively block autophagy. Rats of Control group received no treatment; rats in the VILI group were treated with a ventilator following the protocol described below (Section 2.2 VILI model); rats in the VILI + 3-MA group were intraperitoneally injected with the autophagy inhibitor 3-MA (15 mg/kg, Sigma, St. Louis, MO,USA) 30 min before application of the VILI model; rats in the VILI + CLQ group were intraperitoneally injected with the autophagy inhibitor chloroquine (CLQ; 20 mg/kg, Sigma Chemical) 30 min before application of the VILI model.

### VILI model

Firstly, rats were anesthetized through pentobarbital (50 mg/kg, i.p.) and fentanyl (0.05 mg/kg, i.p.), and laid in a supine position on a pad. The temperature of rats was maintained with a heating lamp. Carotid catheters were then placed to continuously monitor blood pressure. No significant difference was observed in blood pressure among groups. Secondly, the rat was intubated with a catheter (14.0), which was connected to the ventilator (RWD407, RWD Life Science Co., Ltd.). The mechanical ventilation lasted for 4 h under the following parameters: tidal volume, 30 mL/kg; respiratory rate, 50/min; inspiratory/expiratory ratio, 1:1; FiO_2_, 50%. Atracurium (1.5 mg/kg, i.v.) was interval given to maintain muscular; pentobarbital (50 mg/kg, i.p.) and fentanyl (0.05 mg/kg, i.p.) was given to maintain anesthesia. The carotid catheter was used to collect blood sample and monitor the blood pressure using a PowerLab electrophysiolograph instrument (ADInstruments, Bella Vista, Australia). Fluid boluses of Ringer's solution (37°C, 6-12 mL/kg) were given to rats every hour by a jugular catheter to maintain blood pressure.

### Arterial blood gas analysis

As previously described, after rats were anesthetized, carotid catheters were then placed to collect the arterial blood. 1.0 ml of arterial blood was collected from the carotid catheter pre-operatively and immediately after treatment for the Control, Sham, VILI, VILI + H_2_S, and H_2_S groups were finished. Blood gas was analyzed with the Bayer Rapidlab 348 instrument (Bayer Diagnostics, Leverkusen, Germany) to obtain the PaO_2_, PaCO_2_, HCO_3_ and pH value.

### BALF and serum collection

After the treatment was finished, the rats were euthanized with over dose of pentobarbital. The thorax cavity was opened to expose the lung. The right lung was lavaged with saline (5 mL, 4°C) for three times. The collected lavage fluid was centrifuged at 1200 g for 10 min at 4°C. The BALF was tested for total protein and cytokine levels. For serum collection, 1.0 ml of arterial blood was sampled from the carotid catheter. It was kept still for 35 min, and then centrifuged at 1800 g for 20 min. Serum was then collected and analyzed for the levels of biomarkers.

### Histopathological examination

After the rats were euthanized with over dose of pentobarbital, lung tissue was harvested and stained with hematoxylin and eosin (H&E). Three slices were randomly selected from each rat, and five fields of each slice were analyzed by two pathologists uninformed of the experimental grouping under a microscope at a magnification of 200×, as described by Belperio et al^25^. Briefly, the scores were calculated based on the following items: alveolar congestion, hemorrhage, infiltration or aggregation of neutrophils in the airspace and hyaline membrane formation. The scores were from 0 (no injury) to 4 (more than 75% lung was injured). The final score for each group was the average of the scores obtained from all 15 fields.

### Pulmonary edema and permeability measurement

Lung wet-to-dry (W/D) ratio and Evans blue dye leakage were determined as described previously to measure pulmonary edema and permeability^26^-^27^. The right middle lobe of lung of each rat was weighed and dried in an oven at 60°C for 3 days to obtain its dry weight. For Evans blue dye leakage measurement, the Evans blue was injected to each animal (30 mg/kg, i.v.) half an hour before the rats were sacrificed. The lung Evans blue content then collected and measured spectrophotometrically at 620 nm. The final leakage content was calculated based on the standard curve calculated from the optical density at 620 nm. Lung permeability index value was calculated using the Bradford method^27^.

### ELISA assays of inflammatory cytokine expression

The expression levels of interleukin (IL)-1β, IL-6, TNF-α, and MIP-1α in BALF, serum, and lung tissue were measured using the ELISA system (Elabscience, Wuhan, China) according to the manual. The results were determined through spectral scanning plate reader (Varioskan, Thermo Fisher Scientific, Waltham, MA, USA).

### Measurement of oxidative products and anti-oxidative enzymes

The contents of oxidative products (malondialdehyde [MDA], 8-hydroxy-2'-deoxyguanosine [8-OHdG], and protein carbonyl) and anti-oxidative enzymes (catalase [CAT], superoxide dismutase [SOD], and glutathione peroxidase [GPx]) of lung homogenates were detected using the relevant kits according to their manufacturers' instructions. The 8-OHdG ELISA commercial kit was bought from Shanghai Elisa Technology, Ltd. (Shanghai, China). The MDA, protein carbonyl, CAT, SOD, and GPx kits were bought from Nanjing Jiancheng, Co., Ltd. (Nanjing, China).

### Cyclic strain

To investigate which cell types in the alveolar epithelial-endothelial unit might be the source of the observed effects in the lung, cell lines of alveolar epithelial cells (L2 cells), endothelial cells (RAOEC cells), and vascular smooth muscle cells (USMC cells) were bought from Cobioer Biosciences Co., Ltd. (Nanjing, Jiangsu, China). All cells were treated using the cyclic strain (CS) method to mimic the effect of VILI *in vivo*. L2, RAOEC, and USMC cells were plated on culture plates (4 × 10^4^ cells per well). After the cells were grown for 2 days, they were subjected to CS with a FX4000 AFC-CTL Cyclic Stress Unit (Flexcell, Dunn Labortechnik, Asbach, Germany) for 24 h with 20% elongation (1 Hz) applied.

After the cyclic strain was complete, L2, RAOEC, and USMC cells were harvested and the protein levels of Bax, Beclin-1, and GRP78 in cells were measured using western blotting to confirm whether apoptosis, autophagy, or ER stress was induced in these cells. L2, RAOEC, and USMC cells that did not receive cyclic strain treatment were used as controls.

### Treatment of cells with NaHS or 4-PBA (4-Phenylbutyric acid)

L2 cells were cultured in RPMI 1640 medium (10% fetal bovine serum supplemented with 100 units/mL penicillin and100 μg/mL streptomycin). The temperature was kept at 37°C. Confluent cells were randomly assigned into Control, CS, CS + NaHS, and NaHS groups; or Control, CS, CS + 4-phenylbutyric acid (4-PBA), and 4-PBA groups; or Control, CS, CS + NaHS, and CS+4-PBA groups. Cells in the Control received no treatment; cells in the CS group received cyclic strain treatment; cells in the CS + NaHS group were cultured in RPMI 1640 medium with NaHS (100 μM, an H_2_S donor; Sigma, St. Louis, MO, USA) for 24 h and received cyclic strain treatment for 24 h; cells in the NaHS group were cultured in medium containing 100 μM NaHS; and cells in the CS + 4-PBA group were cultured in medium containing 5 mM 4-PBA (Sigma-Aldrich) for 2 h and received cyclic strain treatment for 24 h. The phosphate-buffered saline group was used as a control. After the treatments were complete, L2 cells were harvested and the protein levels of target genes in cells were measured to investigate the involvement of autophagy, ER stress, and the NF-κB/MAPK signaling pathway.

### Western blot

Briefly, the right lung was harvested and placed in ice-cold homogenizing buffer and then homogenized in a 15-mL glass homogenizer. The homogenate was centrifuged at 15000 × *g* for 10 min at 4°C to collect the supernatant. The sediment was oscillated with nucleoprotein extraction buffer and centrifuged at 16000 × *g* for 10 min at 4°C to collect the nuclear protein. The protein concentration was measured using a BCA protein assay kit (Beyotime, Shanghai, China). The protein were separated and transferred to polyvinylidene fluoride membranes. After incubation with primary antibodies (Santa Cruz, CA, USA), they were incubated with secondary antibody (Santa Cruz Biotechnology, Santa Cruz, CA, USA). Bands were visualized with enhanced chemiluminescence method. The bar graphs show average values quantified from the optical densities of six bands for each group.

### Statistical analysis

Statistical analysis was carried out with the SPSS software version 19 (SPSS Inc., Chicago, IL, USA) using one-way analysis of variance (one-way ANOVA). Testing for normality showed that data had normal distribution. The Bonferroni post-hoc test was performed afterwards. When p value<0.05, the difference was considered significant.

## 3. Results

### Changes in physiological parameters with VILI or H_2_S treatment

Table 1 shows the physiological parameters of rat arterial blood (PaO_2_, PaCO_2_, HCO_3_, and pH) in different groups measured pre- and post-operatively. There was no significant difference in pre-operative measurements of these parameters among groups. Post-operatively, compared to the Control, PaO_2_ and pH were significantly lower, while PaCO_2_ and HCO_3_ were significantly higher in the VILI group. Compared to the VILI group, PaO_2_ and pH were significantly higher in the VILI + H_2_S group, while PaCO_2_ and HCO_3_ were significantly lower. These results demonstrated that the respiratory function of rats was significantly impaired with VILI model application, and that H_2_S treatment could partly restore it.

### Changes in histopathological appearance, lung edema, and permeability

**Figure 1:**
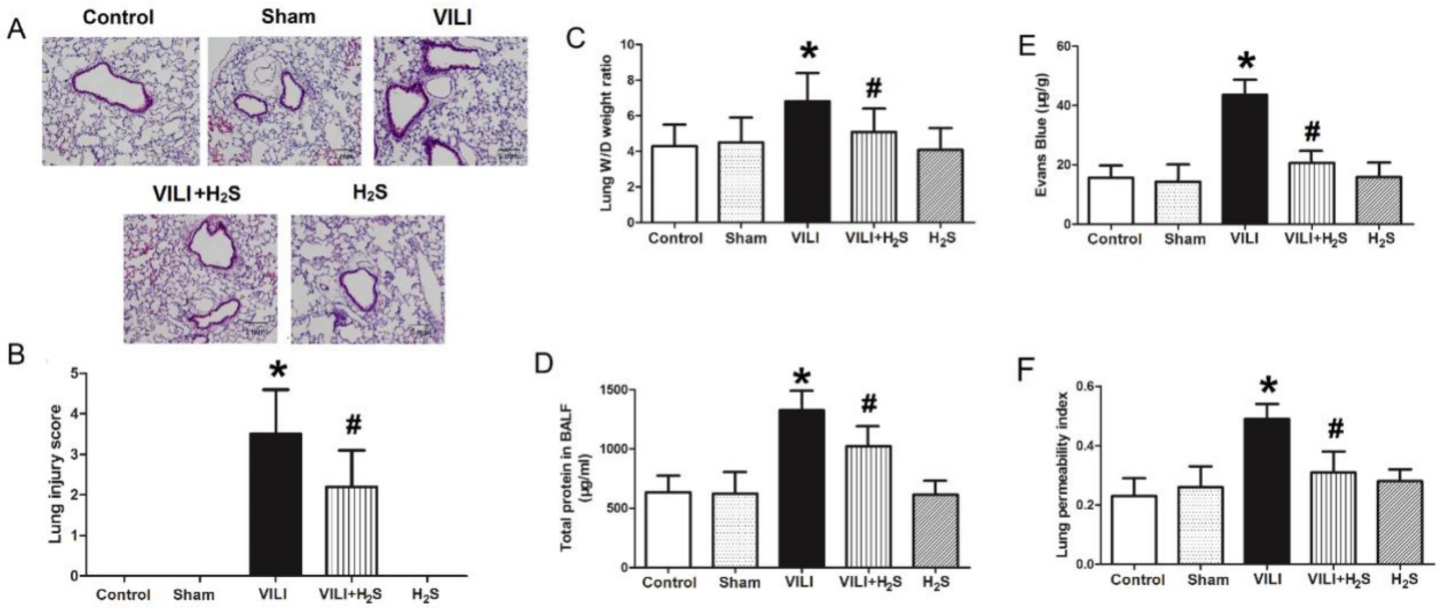
** Changes in histopathological appearance, lung edema, and permeability. A)** images of H&E stained tissue; **B)** lung injury scores; **C)** lung wet-to-dry weight ratio; **D)** Evans blue leakage amount; **E)** lung permeability index; **F)** total protein levels in BALF. H&E: hematoxylin and eosin; BALF: bronchoalveolar lavage fluid. *: p < 0.05 compared to Control; #: p < 0.05 compared to VILI. n = 10.

Figure 1 shows changes in histopathological appearance, lung edema, and permeability. As shown in Figure 1A (H&E staining), in the VILI group, the lung tissues demonstrated thickening of the alveolar septa and infiltration of inflammatory cells. After rats were treated with H_2_S, the thickening of alveolar septa and inflammatory cell infiltration decreased significantly. Figure 1B summarizes the lung injury scores calculated from Figure 1A. The lung injury score of the VILI group was significantly higher than that of the Control, although H_2_S treatment could reduce it somewhat. As shown in Figure 1C-F, the lung W/D weight ratio, Evans blue staining, lung permeability index, and total protein levels in BALF of the VILI group were markedly increased compared to the Control. H_2_S treatment significantly decreased these parameters compared to VILI. Sham or H_2_S treatment alone did not significantly change these parameters. These results indicated that structural damage was caused by the VILI model, which could be attenuated with H_2_S treatment.

### Changes in pro- and anti-inflammatory factors in BALF, serum, and lung tissue

**Figure 2:**
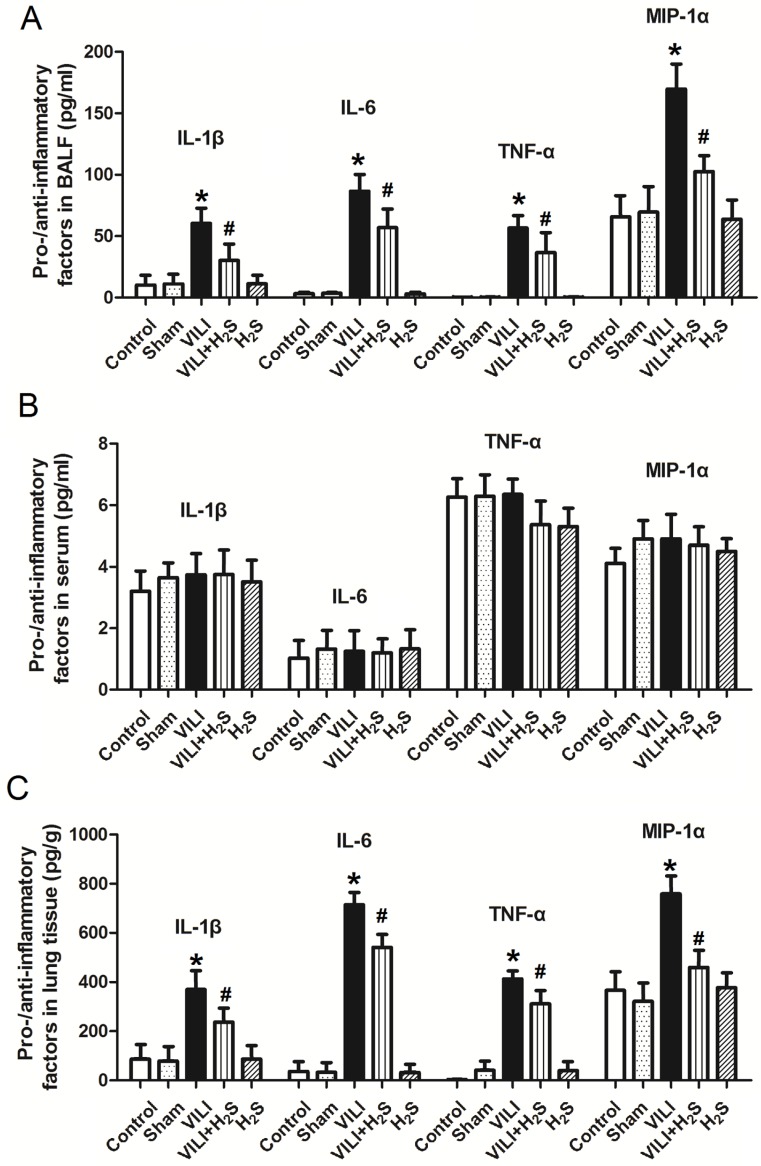
** Changes in pro- and anti-inflammatory factors (IL-1β, IL-6, TNF-α, and MIP-1α) in BALF, serum, and lung tissue.** A: levels of pro- and anti-inflammatory factors in BALF; B: levels of pro- and anti-inflammatory factors in serum; C: levels of pro- and anti-inflammatory factors in lung tissue. BALF: bronchoalveolar lavage fluid; IL-1β: interleukin-1β; IL-6: interleukin-6, TNF-α: tumor necrosis factor-α; MIP-1α: macrophage inflammatory protein-1α. *: p < 0.05 compared to Control; #: p < 0.05 compared to VILI. n = 10.

Figure 2 shows the changes in pro- and anti-inflammatory factors in BALF, serum, and lung tissue. As shown in Figure 2A, the levels of IL-1β, IL-6, TNF-α, and MIP-1α in BALF of the VILI group were much higher than those of the Control. Compared to VILI, H_2_S treatment significantly decreased these parameters. Sham or H_2_S treatment alone did not significantly change these parameters. As shown in Figure 2B, there were no significant differences in the serum levels of IL-1β, IL-6, TNF-α, or MIP-1α among groups. As shown in Figure 2C, the levels of IL-1β, IL-6, TNF-α, and MIP-1α in lung tissue of the VILI group were significantly higher than those of the Control. H_2_S treatment significantly decreased these parameters compared to VILI. Sham or H_2_S treatment alone did not significantly change these parameters. In summary, the VILI model caused significant inflammation in the lung tissue, which could be attenuated with H_2_S treatment.

### Changes in apoptotic proteins in lung tissue

**Figure 3:**
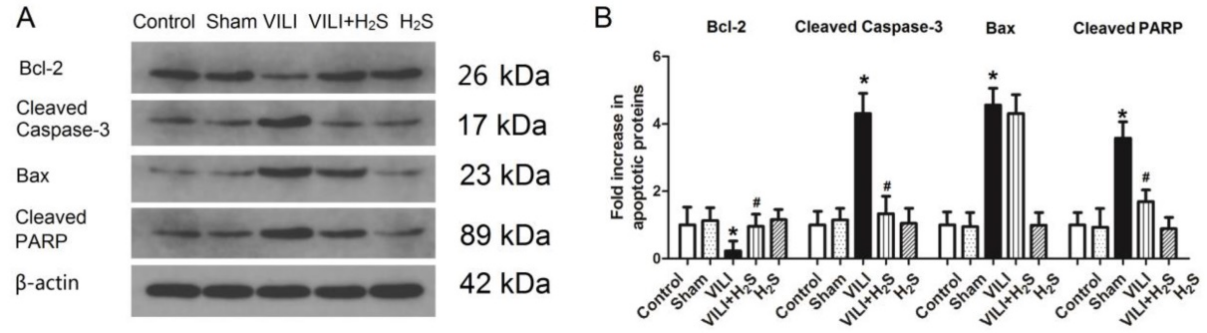
** Changes in apoptotic proteins (Bcl-2, cleaved Caspase-3, Bax, and cleaved PARP) in lung tissue. A)** representative bands from western blot analysis; **B)** levels of apoptotic proteins in lung tissue. Bcl-2: B-cell lymphoma-2; Bax: Bcl-2 associated X protein; cleaved PARP: cleaved poly ADP-ribose polymerase. *: p < 0.05 compared to Control; #: p < 0.05 compared to VILI. n = 6.

Figure 3 shows changes in apoptotic proteins (Bcl-2, cleaved Caspase-3, Bax, and cleaved poly ADP-ribose polymerase (PARP)) in lung tissue. The protein level of Bcl-2, an apoptosis inhibiting protein, was much lower in the VILI group than in the Control group. H_2_S treatment significantly increased the protein level of Bcl-2 compared to VILI. Sham or H_2_S treatment alone did not significantly change the protein level of Bcl-2. The protein levels of cleaved Caspase-3, Bax, and cleaved PARP, three apoptosis promoting proteins, were significantly elevated in the VILI group compared with the Control group. H_2_S treatment significantly decreased the protein levels of cleaved Caspase-3, Bax, and cleaved PARP compared to VILI. Sham or H_2_S treatment alone did not significantly change these protein levels. Taken together, these results revealed that the VILI model induced cellular apoptosis in the lung, which could be inhibited by H_2_S treatment.

### Changes in oxidative products and anti-oxidative enzymes in the lung

**Figure 4:**
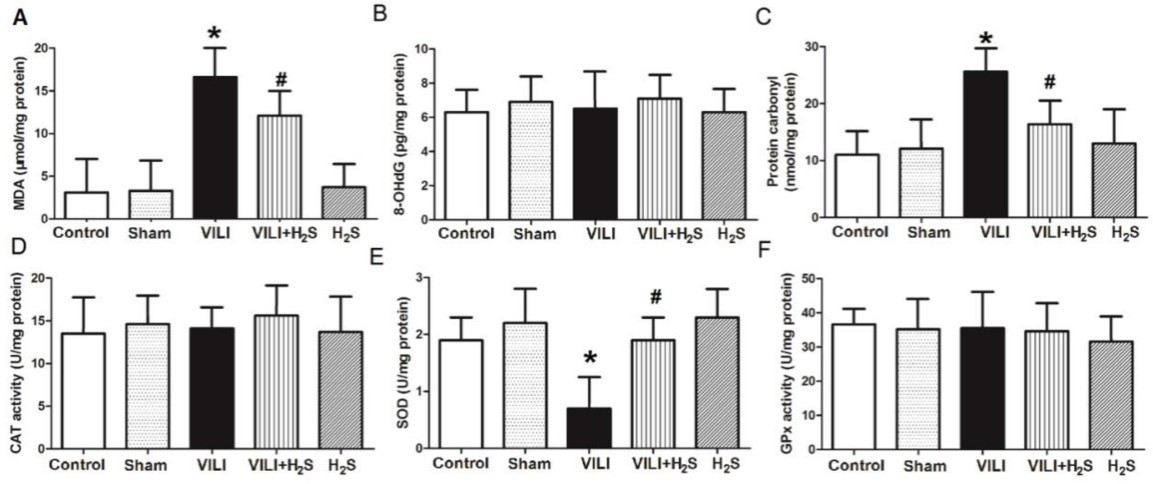
** Changes in oxidative products and anti-oxidative enzymes in lung tissue. A)** MDA levels; **B)** 8-OHdG levels; **C)** protein carbonyl levels; **D)** CAT levels; **E)** SOD levels;** F)** GPx levels. MDA: malondialdehyde; 8-OHdG: 8-hydroxy-2'-deoxyguanosine; CAT: catalase; SOD: superoxide dismutase; GPx: glutathione peroxidase. *: p < 0.05 compared to Control; #: p < 0.05 compared to VILI. n = 10.

Figure 4 shows changes in oxidative products (MDA, 8-OHdG, and protein carbonyl) and anti-oxidative enzymes (CAT, SOD, and GPx) in the lung. As shown in Figure 4A and C, the levels of MDA and protein carbonyl in the lungs of the VILI group were significantly increased compared to Control, and H_2_S significantly decreased their levels compared to VILI. Sham or H_2_S treatment alone did not significantly change these parameters. The levels of 8-OHdG showed no significant difference among groups, indicating that the VILI model did not induce oxidation of DNA.

As shown in Figure 4E, the level of SOD in the lungs of the VILI group was much lower than that of the Control group. H_2_S treatment significantly ameliorated the decrease of SOD caused by VILI. Sham or H_2_S treatment alone did not significantly change these parameters. The levels of CAT and GPx in the lung showed no significant differences among groups. These results suggest that SOD was the main anti-oxidative enzyme consumed in the VILI model, and that H_2_S treatment could significantly restore it.

### Changes in mTOR activation and the autophagy proteins p62, Beclin-1, and p-S6 in lung tissue

**Figure 5:**
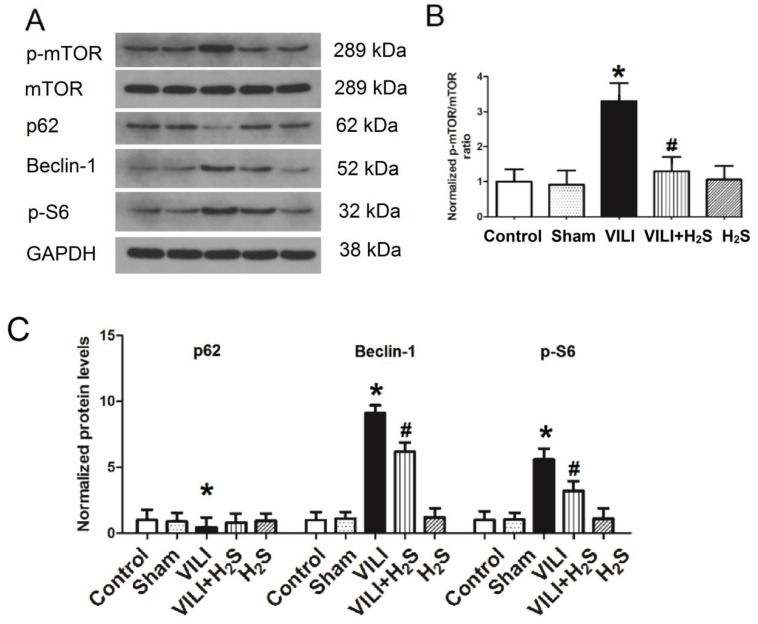
** Changes in mTOR activation and autophagy proteins (p62, Beclin-1, and p-S6) in lung tissue. A)** representative bands from western blot analysis; **B)** phosphorylation level of mTOR; **C)** levels of autophagy proteins (p62, Beclin-1, and p-S6). mTOR: mammalian target of rapamycin. *: p < 0.05 compared to Control; #: p < 0.05 compared to VILI. n = 6.

Figure 5 shows the phosphorylation of mTOR and autophagy proteins (p62, Beclin-1, and p-S6) in lung tissue. As shown in Figure 5A-C, the phosphorylation level of mTOR and the expression of Beclin-1 and p-S6, two autophagy promoting proteins, all increased significantly in the VILI group compared to the Control. H_2_S treatment significantly decreased their levels compared to VILI. The expression of p62, an autophagy inhibiting protein, was significantly lower in the VILI group than in the Control, but increased with H_2_S treatment compared to VILI. These results indicated that autophagy was significantly activated by the VILI model, but partially inhibited with H_2_S treatment.

### Effects of autophagy inhibitors on the severity of VILI

**Figure 6:**
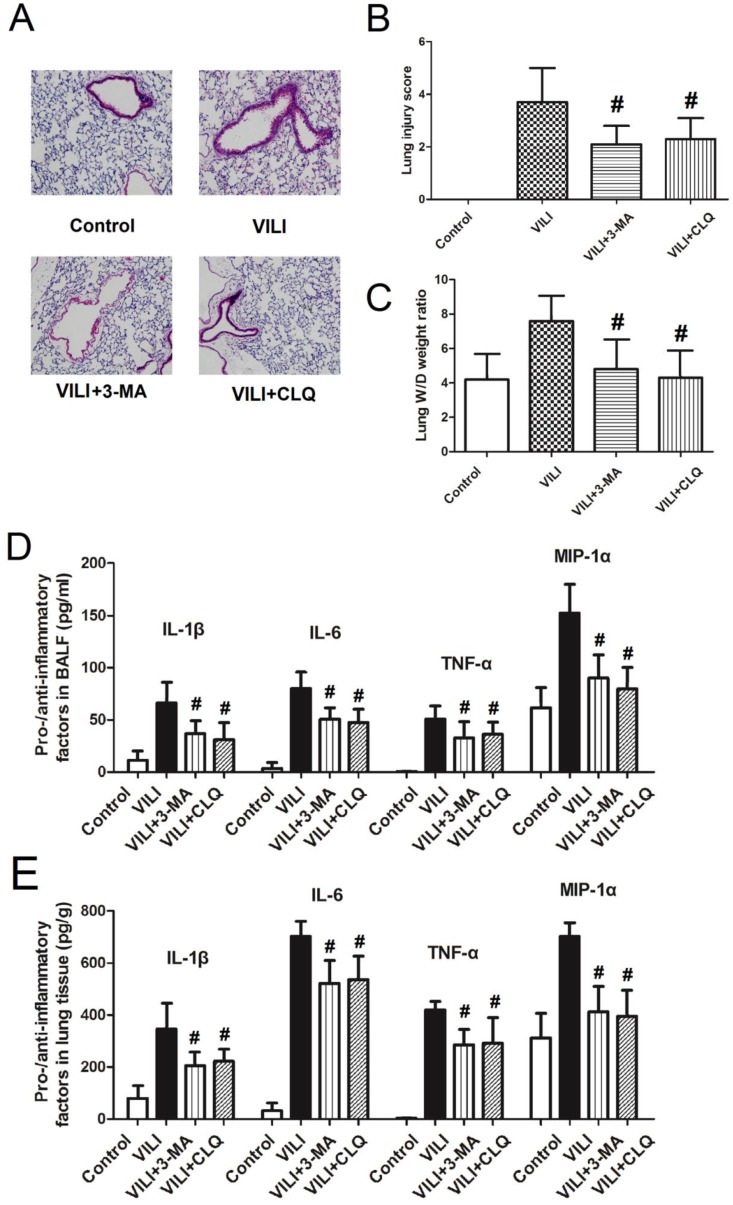
** Effects of autophagy inhibitors (3-MA and CLQ) on the severity of ventilator-induced lung injury. A)** lung H&E staining results; **B)** lung injury score; **C)** lung wet-to-dry weight ratio; **D)** levels of pro- and anti-inflammatory factors in BALF; **E)** levels of pro- and anti-inflammatory factors in lung tissue. H&E: hematoxylin and eosin; BALF: bronchoalveolar lavage fluid; 3-MA: 3-methyladenine; CLQ: chloroquine. #: p < 0.05 compared to VILI. n = 10.

To confirm the role of autophagy in the development of VILI, we tested the effects of autophagy inhibitors on the severity of VILI, as shown in Figure 6. Figure 6A shows that after rats were treated with autophagy inhibitors (3-MA and CLQ), thickening of alveolar septa and inflammatory cell infiltration decreased significantly. Figure 6B shows that the lung W/D weight ratio decreased sharply following treatment with 3-MA or CLQ. Figure 6C and D show changes in pro- and anti-inflammatory factors in BALF and lung tissue. Both 3-MA and CLQ treatment led to significant decreases in the levels of IL-1β, IL-6, TNF-α, and MIP-1α in BALF and lung tissue. In summary, autophagy inhibition significantly decreased the severity of VILI.

### Induction of apoptosis, autophagy, and ER stress in various cell lines treated with cyclic strain

**Figure 7:**
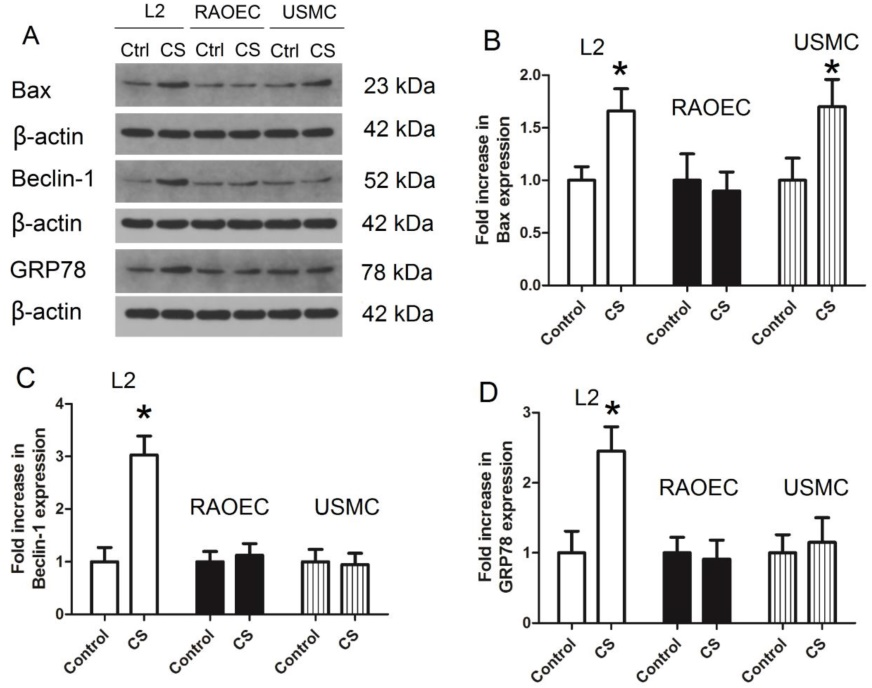
** Protein levels of Bax, Beclin-1, and GRP78 in L2, RAOEC, and USMC cells treated with cyclic strain. A)** representative bands from western blot analysis; **B)** levels of Bax; **C)** levels of Beclin-1; **D)** levels of GRP78. CS: cyclic strain; GRP78: glucose-regulated protein 78; L2 cells: rat alveolar epithelial cell line; RAOEC cells: rat aortic endothelial cell line; USMC cells: rat vascular smooth muscle cell line. *: p < 0.05 compared to Control; #: p < 0.05 compared to VILI. n = 6.

To investigate which cell types in the alveolar epithelial-endothelial system might be responsible for the effect observed in the rat VILI model, L2, RAOEC, and USMC cells were treated with cyclic strain to mimic the mechanical strain, and the protein levels of Bax, Beclin-1, and GRP78 were measured in these cells to confirm whether apoptosis, autophagy, or ER stress were induced under cyclic strain. As shown in Figure 7, protein expression of Bax increased significantly in both L2 and USMC cells; the expression of Beclin-1 and GRP78 increased in L2 cells. These findings suggested that apoptosis, autophagy, and ER stress were induced in L2 cells following treatment with cyclic strain. Therefore, L2 cells were used in subsequent experiments.

### Changes in ER stress proteins and activation of ER stress-related pathways in L2 cells treated with cyclic strain

**Figure 8:**
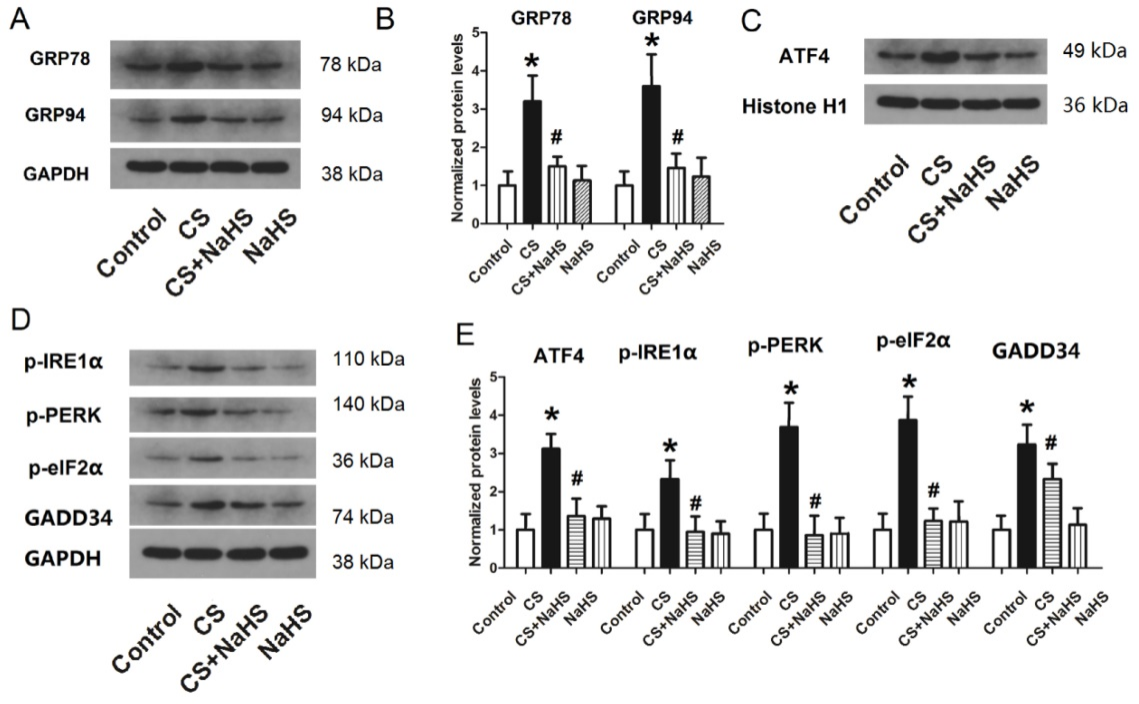
** Changes in ER stress-related proteins in L2 cells. A)** representative bands of GRP78 and GRP94 from western blot analysis; **B)** protein levels of GRP78 and GRP94 in L2 cells; **C)** representative bands of nuclear ATF4 from western blot analysis; **D)** representative bands of p-IRE1α, p-PERK, p-eIF2α, and GADD34 from western blot analysis; **E)** protein levels of nuclear ATF4, p-IRE1α, p-PERK, p-eIF2α, and GADD34 in L2 cells. ER: endoplasmic reticulum; GRP: glucose-regulated protein; ATF4: activating transcription factor 4; IRE1α: α subunit of inositol-requiring enzyme; PERK: protein kinase RNA-like ER kinase; eIF2α: α subunit of eukaryotic translation initiation factor 2; GADD34: growth arrest and DNA damage-inducible gene 34; *: p < 0.05 compared to Control; #: p < 0.05 compared to VILI. n = 6.

To explore the mechanism of the effect of NaHS on cyclic strain, we measured changes in ER stress and activation of ER stress-related pathways (nuclear ATF4, p-IRE1α, p-PERK, p-eIF2α, and GADD34) due to cyclic strain or NaHS treatment. As shown in Figure 8A and B, the protein levels of two ER stress proteins, GRP78 and GRP94, increased sharply in the CS group, but decreased significantly with NaHS treatment. Figure 8C-E shows the level of nuclear ATF4 and phosphorylation levels of IRE1α, PERK, and eIF2α and GADD34 associated with CS or NaHS treatment. The protein levels of p-IRE1α, p-PERK, p-eIF2α, nuclear ATF4, and GADD34 all increased significantly in the CS group, and decreased significantly with NAHS treatment compared to CS. NaHS alone had no effect on ER stress proteins or ER stress-related pathways (nuclear ATF4, p-IRE1α, p-PERK, p-eIF2α, and GADD34). Taken together, these data indicated that CS induced ER stress, whereas NaHS treatment could attenuate it.

### Changes in autophagy proteins and the NF-κB/MAPK pathway with 4-PBA or NaHS

**Figure 9:**
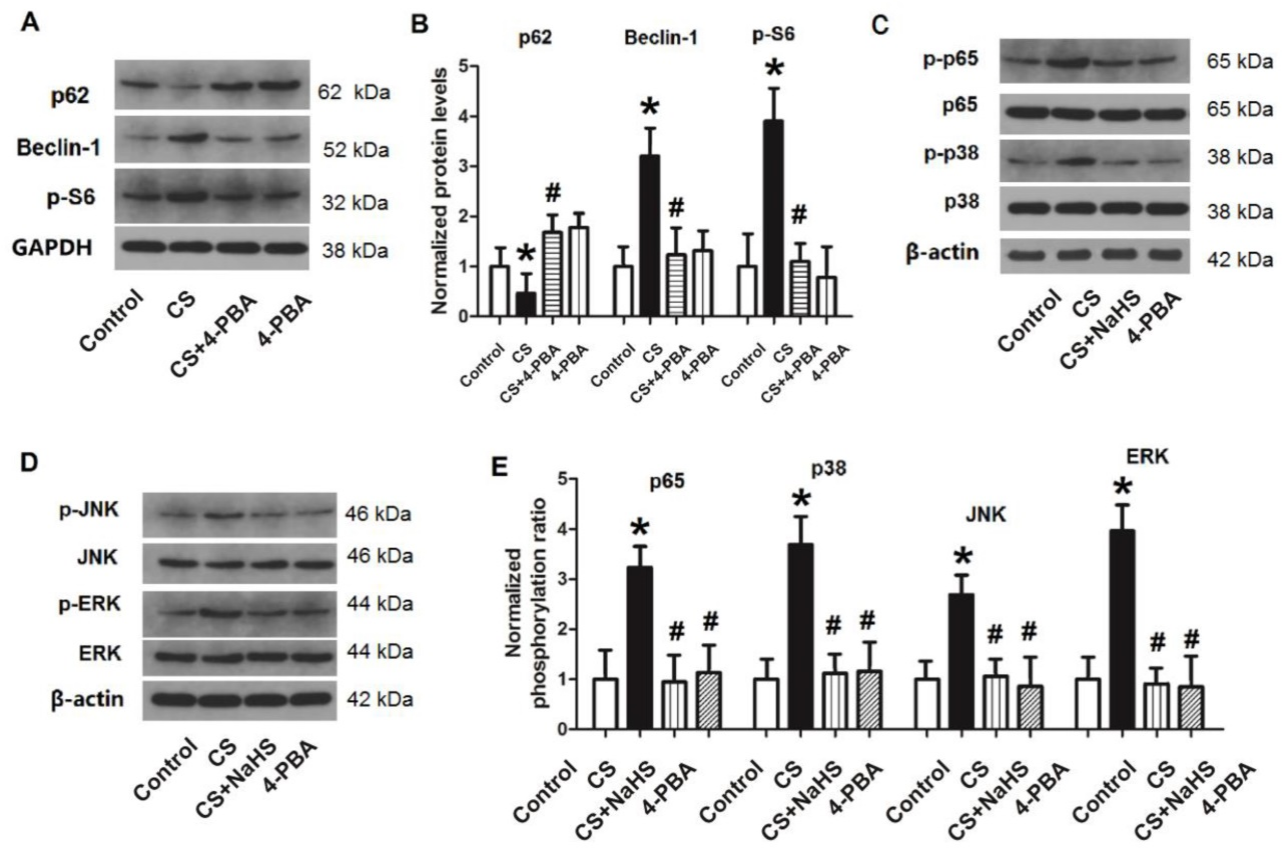
** Changes in autophagy proteins and the NF-κB/MAPK pathway in L2 cells.** After L2 cells were treated with 4-PBA or NaHS, the protein levels of autophagy proteins and the NF-κB/MAPK pathway were measured through western blotting. **A)** representative bands of autophagy proteins (p62, Beclin-1, and p-S6) from western blot analysis; **B)** levels of autophagy proteins (p62, Beclin-1, and p-S6); **C)** representative bands of nuclear p-p65 and p-p38 from western blot analysis; **D)** representative bands of p-JNK and p-ERK from western blot analysis; **E)** phosphorylation ratio of nuclear p65 (p-p65/p65), p38 (p-p38/p38), JNK (p-JNK/JNK), and ERK (p-ERK/ERK). NF-κB: nuclear factor κB; MAPK: mitogen-activated protein kinases; 4-PBA: 4-phenylbutyrate; JNK: c-Jun-N-terminal kinase; ERK: extracellular signal-regulated kinase; *: p < 0.05 compared to Control; #: p < 0.05 compared to VILI. n = 6.

As shown in Figure 9A and B, following treatment of L2 cells with the ER stress inhibitor 4-PBA, the protein level of p62 increased significantly compared to those treated with CS. By contrast, the protein levels of Beclin-1 and p-S6 were significantly lower than with cyclic strain. These results suggested that inhibition of ER stress could inhibit cellular autophagy in the CS group. In other words, cyclic strain may cause cellular autophagy through activation of ER stress.

Next, we treated cells with CS + 4-PBA or CS + NaHS, and measured the phosphorylation levels of nuclear NF-κB p65, MAPK p38, JNK, and ERK. As shown in Figure 9C-E, nuclear NF-κB p65, MAPK p38, JNK, and ERK were all activated in the CS group (p < 0.05). After rats were treated with 4-PBA or NaHS, the phosphorylation levels of these proteins all decreased significantly compared to the CS group. These results revealed that CS activates the NF-κB/MAPK signaling pathway, which can be inhibited by NaHS or the ER stress inhibitor 4-PBA. Thus, ER stress may activate the NF-κB/MAPK signaling pathway, while H_2_S may inhibit this pathway by inhibiting ER stress.

## Discussion

Reported here is an investigation into the effect and mechanism of H_2_S treatment of VILI using both *in vivo* and *in vitro* studies. The protective effect of H_2_S treatment against VILI was demonstrated by the analysis of respiratory function indicators, lung tissue histopathology changes, oxidative stress parameters (MDA, protein carbonyl, and SOD), inflammatory factors (IL-1β, IL-6, TNF-α, and MIP-1α), and apoptotic proteins (Bcl-2, Caspase-3, Bax, and cleaved PARP). Further experiments indicated that autophagy and ER stress were involved in the development of VILI, and that H_2_S treatment could inhibit these processes. The *in vitro* experiments showed that H_2_S treatment might inhibit autophagy and ER stress in lung alveolar epithelial cells through regulation of the PERK/eIF2α/ATF4/GADD34 pathway. Taken together, these findings showed that H_2_S treatment could attenuate the degree of VILI through inhibition of autophagy and ER stress in alveolar epithelial cells and that the PERK/eIF2α/ATF4/GADD34 pathway was involved.

In the present study, the levels of PaO_2_, PaCO_2_, HCO_3_, and pH in arterial blood changed significantly with the VILI model, indicating that respiratory function was impaired by VILI. Thickening of the alveolar septa and infiltration of inflammatory cells, as well as increases in the lung W/D weight ratio, Evans blue leakage, lung permeability index, and total protein levels in BALF of the VILI group indicated that lung tissue was damaged in VILI. Furthermore, the levels of oxidative stress parameters (MDA, protein carbonyl, and SOD), inflammatory factors (IL-1β, IL-6, TNF-α, and MIP-1α), and apoptotic proteins (Bcl-2, Caspase-3, Bax, and cleaved PARP) in the VILI group were all significantly higher than those of the Control, suggesting the induction of inflammation, oxidative stress, and apoptosis in VILI. Taken together, these results showed that VILI caused an inflammatory cascade reaction, resulting in pulmonary edema, airway shaping, airway obstruction, and imbalance of ventilation with blood flow. These findings are in accordance with the pathological mechanism of ventilator-associated lung injury, which indicates the success of the VILI model established in the study.

The effect of H_2_S is related to its source and concentration. Previous research has shown that H_2_S at 80 ppm can inhibit the endotoxin-induced systemic inflammatory response^7^, reduce acute lung injury, and improve survival rate in mice^28^. A large number of studies have confirmed that H_2_S has important pathophysiological roles in various systemic and local inflammatory reactions. Inhalation of 80 ppm H_2_S for 6 h has shown anti-oxidative and anti-inflammatory effects in other mouse models of lung injury^3^,^5^. Therefore, inhalation of 80 ppm H_2_S was chosen for the present study. The results of arterial blood gas analysis, H&E staining, lung W/D weight ratio, Evans blue leakage, lung permeability index, and total protein levels in BALF showed that treatment with 80 ppm H_2_S could effectively attenuate the degree of VILI. H_2_S treatment maintained the pH balance of the arterial blood while preventing damage to lung tissue and the disruption of lung permeability.

The mechanism of action of H_2_S and the target of its effects can vary widely. In recent years, studies have confirmed that H_2_S regulates the inflammatory response^3^ and promotes vasodilatation^6^; it also exhibits anti-oxidative stress^5^ and anti-fibrosis effects. Exogenous H_2_S provided as intravenous sodium hydrosulfide has been confirmed to have protective effects in various animal studies of ALI/ARDS due to its anti-inflammatory, anti-oxidative, and anti-apoptotic properties^5^. In the endotoxin-induced ALI mouse model^7^,^28^, H_2_S inhibited the systemic inflammatory response and improved the survival rate in mice; in hyperventilation-induced ALI, H_2_S could inhibit the intrapulmonary inflammatory response and alveolar epithelial cell apoptosis, thus mitigating lung injury^28^. Consistent with these previous findings, the present study demonstrated that H_2_S treatment significantly decreased the inflammatory response of rats. These results suggested that H_2_S treatment could interfere with the inflammatory reaction in rats with VILI and reduce pro-inflammatory factors in BALF and lung tissue. Furthermore, H_2_S treatment significantly decreased the protein level of Bcl-2 and also decreased the protein levels of cleaved Caspase-3, Bax, and cleaved PARP compared to VILI, suggesting that it effectively inhibited cell apoptosis in the lung. MDA is a product created from lipid peroxidation of ROS and phospholipids in the biomembrane or membrane receptor-related polyunsaturated fatty acid side chains. Protein carbonyl, on the other hand, measures the oxidation of protein structures. The increased MDA and protein carbonyl levels indicated that the VILI model induced oxidation of the lipid and protein structures of cells. H_2_S treatment significantly lowered the levels of MDA and protein carbonyl compared to VILI and restored the level of SOD, indicating the effects of H_2_S on apoptosis and oxidative stress. Thus, H_2_S treatment might inhibit the release of cytokines, reducing protein exudation in the alveolar space, neutrophil infiltration, apoptosis, and oxidative stress in lung tissue, thereby improving ventilation, increasing arterial oxygen partial pressure, and significantly reducing lung damage. However, the mechanism underlying this process requires further exploration.

Due to the complex etiology and predisposing factors of ALI, there is currently no consistent understanding of the role of autophagy in the development of ALI. To explore the role of autophagy in VILI, the present study examined the phosphorylation of mTOR and autophagy proteins (p62, Beclin-1, and p-S6) in lung tissue, then measured the effects of autophagy inhibitors (3-MA and CLQ) on the severity of VILI. The results revealed that the phosphorylation level of mTOR and the expression of Beclin-1 and p-S6 in the VILI group increased significantly compared to Control. After rats were treated with 3-MA or CLQ, the degree of thickening of alveolar septa and inflammatory cell infiltration decreased significantly. The lung W/D weight ratio as well as pro- and anti-inflammatory factors (IL-1β, IL-6, TNF-α, and MIP-1α) in BALF and lung tissue were decreased significantly following treatment with 3-MA and CLQ. Taken together, these results indicated that autophagy was activated in the VILI model and played an important role in VILI.

Several cell types in the alveolar epithelial-endothelial unit may be the source of the effects observed in the lung, including alveolar epithelial cells, endothelial cells, and smooth muscular cells. To verify which cell types were involved, we treated L2, RAOEC, and USMC cells with cyclic strain to mimic the mechanical strain, then measured the protein levels of Bax, Beclin-1, and GRP78 to confirm induction of apoptosis, autophagy, and ER stress in these cells. Only the L2 cells exhibited apoptosis, autophagy, and ER stress. These findings suggested alveolar epithelial cells might be the source of the effects observed in the lung with the VILI model. Therefore, L2 cells were used to further explore the mechanism of autophagy and ER stress.

ER stress has shown been shown to both activate and inhibit autophagy. UPR triggered by ER stress can induce autophagy through regulation of Akt1-mTOR, AMPK, or alteration of ER Ca^2+^, and may inhibit AMPK to prevent autophagy. ER stress and autophagy share functional characteristics and may also interact. To confirm the effect of H_2_S on ER stress in L2 cells, we treated L2 cells with cyclic strain and the H_2_S donor NaHS, and measured the expression of two ER stress proteins, GRP78 and GRP94. When the ER is functional and stress-free, the receptor molecules ATF6 and PERK in the UPR signaling pathway are bound to the GRP chaperones GRP78 and GRP94 located on the ER, and enter a non-activated state 22. GRPs are important markers of ER stress and can be significantly elevated in the presence of ER stress. GRP78 promotes protein refolding, modification, and oligomerization in the ER to attain the correct structure. GRPs were highly elevated in the CS group, but significantly decreased following NaHS treatment. These results indicated that ER stress could be induced by cyclic strain, and that H_2_S could significantly inhibit ER stress in L2 cells.

A growing body of research has shown that ER stress can induce autophagy, and in most cases, the unfolded protein-reactive PERK/eIF2α/ATF4/GADD34 signaling pathway is activated^29^. When PERK and GRP78 dissociate, autophosphorylation occurs and causes phosphorylation of eIF2α, thus reducing the synthesis of most proteins in the cell. Phosphorylated eIF2α can lead to overexpressed ATF4 entering the nucleus, resulting in increased transcription of downstream molecules such as GADD34. To explore the mechanism of the effect of H_2_S on VILI, we measured changes in the PERK/eIF2α/ATF4/GADD34 pathway in L2 cells treated with cyclic strain and H_2_S. The results demonstrated that expression of p-IRE1α, p-PERK, p-eIF2α, nuclear ATF4, and GADD34 all increased significantly with cyclic strain, but were significantly repressed by H_2_S treatment. These results indicated that ER stress and autophagy were involved in mechanical strain through the PERK/eIF2α/ATF4/GADD34 pathway, and that H_2_S treatment could inhibit ER stress and autophagy by inhibiting this pathway.

Recent studies have confirmed that 4-PBA acts as a chemical chaperone, inhibiting ER stress and UPR responses. For example, 4-PBA was found to maintain blood homeostasis in type 2 diabetic mice by reducing the effects of ER stress and to improve the leptin response in the hypothalamus of obese mice by inhibiting UPR responses^30^. To explore the effects of ER stress on autophagy, we treated L2 cells with cyclic strain and the ER stress inhibitor 4-PBA, then measured the expression of autophagy proteins (p62, Beclin-1, and p-S6). The results indicated that 4-PBA successfully inhibited autophagy in cells, suggesting that cyclic strain might induce autophagy in cells by activating ER stress. ER stress is an important cause of inflammation, but the mechanism through which the stress response leads to the inflammatory response has not been clearly elucidated. IRE1, an ER transmembrane sensor, could activate the UPR and maintain ER and cellular functions^31^. The IRE1α-TRAF2 complex can recruit IκB kinase (IKK) to phosphorylate IκB, which dissociates NF-κB from the NF-κB-IκB complex. NF-κB can translocate to the nucleus, thereby initiating transcription of inflammatory genes^32^. ER stress-induced NF-κB activation and inflammatory factor TNF-α production are significantly reduced in IRE1α knockout mouse embryonic fibroblasts^33^. PERK and eIF2α can also regulate NF-κB activation. Because the half-life of IκB is significantly shorter than that of NF-κB, PERK-eIF2α-mediated translational inhibition increases the ratio of NF-κB/IκB, releasing “excess” NF-κB into the nucleus and promoting inflammation.

MAPKs are highly conserved serine protein kinases in the cytoplasm, and include extracellular signal-regulated kinase (ERK), C-Jun amino-terminal kinase (JNK), and p38 MAPK 34. IRE1α can regulate the MAPK protein JNK. Recent studies have shown that activation of the PERK and IRE1 signaling pathways under ER stress can also activate the JNK signaling pathway. ATF4 in the PERK signaling pathway can upregulate inflammatory cytokines by activating the JNK signaling pathway 35. To explore the role of the NF-κB/MAPK pathway in ER stress regulation, we measured the phosphorylation levels of nuclear NF-κB p65, MAPK p38, JNK, and ERK after L2 cells were treated with cyclic strain and NaHS or 4-PBA. These results revealed that the NF-κB/MAPK signaling pathway was activated by cyclic strain, while treatment with NaHS or 4-PBA, inhibitors of ER stress, could effectively prevent activation of the NF-κB/MAPK signaling pathway, indicating the involvement of ER stress in activation of the NF-κB/MAPK signaling pathway in the development of VILI. In accordance with these claims, the present study suggested that IRE1α was involved in the development of VILI.

In conclusion, the present study revealed that H_2_S treatment alleviated VILI through regulation of autophagy and ER stress. H_2_S treatment could reduce histopathological impairment, lung edema and permeability, inflammation, apoptosis, and oxidative injury in VILI. Further investigation found that H_2_S reduced autophagy and ER stress induced by VILI or cyclic strain. The PERK/eIF2α/ATF4/GADD34 and NF-κB/MAPK signaling pathways were found to be involved in the underlying mechanism, adding a new dimension to our understanding of the biological effects of H_2_S. The present research on the protective effect and mechanism of H_2_S on VILI provides an experimental basis for its use and supports its potential value for clinical application.

## Figures and Tables

**Table 1:** Changes of physiological parameters in arterial blood

	Control	Sham	VILI	VILI+H_2_S	H_2_S
**Pre-operation**					
PaO_2_(mmHg)	91.5±5.8	92.5±3.9	89.1±4.8	93.2±5.5	87.9±5.3
PaCO_2_(mmHg)	40.5±4.9	42.8±4.7	43.1±4.2	46.5±4.8	41.9±4.1
HCO_3_(mmol/L)	23.6±4.2	22.9±3.5	24.1±3.3	25.2±3.7	24.6±3.2
pH	7.41±0.06	7.37±0.05	7.36±0.04	7.38±0.05	7.36±0.04
**Post-operation**					
PaO_2_(mmHg)	90.4±6.5	89.6±5.2	61.6±6.1^#^	80.6±5.7*	91.4±7.2
PaCO_2_(mmHg)	41.8±3.9	40.3±3.3	49.9±3.3^#^	44.6±3.5*	42.5±3.6
HCO_3_(mmol/L)	22.5±3.2	21.9±2.8	17.3±2.1^#^	20.4±2.3*	22.3±2.5
pH	7.37±0.05	7.31±0.06	7.21±0.03^#^	7.33±0.04*	7.41±0.03
